# Mucosal Microbiota and Metabolome in the Ileum of Hu Sheep Offered a Low-Grain, Pelleted or Non-pelleted High-Grain Diet

**DOI:** 10.3389/fmicb.2021.718884

**Published:** 2021-08-26

**Authors:** Ruiyang Zhang, Zhiqiang Zhong, Huiting Ma, Limei Lin, Fei Xie, Shengyong Mao, David M. Irwin, Zhe Wang, Shuyi Zhang

**Affiliations:** ^1^College of Animal Science and Veterinary Medicine, Shenyang Agricultural University, Shenyang, China; ^2^Laboratory of Gastrointestinal Microbiology, Jiangsu Key Laboratory of Gastrointestinal Nutrition and Animal Health, College of Animal Science and Technology, Nanjing Agricultural University, Nanjing, China; ^3^Department of Laboratory Medicine and Pathobiology, University of Toronto, Toronto, ON, Canada

**Keywords:** Hu sheep, high-grain diet, microbiota, metabolome, ileal mucosa, probiotics

## Abstract

Alterations in mucosal microbiota and metabolites are critical to intestinal homeostasis and host health. This study used a combination of 16S rRNA gene sequencing and liquid chromatography-mass spectrometry (LC/MS) to investigate mucosal microbiota and their metabolic profiles in the ileum of Hu sheep fed different diets. Here, we randomly allocated 15 Hu sheep to three diets, a non-pelleted low-grain diet (control diet; CON), a non-pelleted high-grain diet (HG), and a pelleted high-grain diet (HP). After 60 days of treatment, ileal mucosal samples were collected for microbiome and metabolome analysis. The results of principal coordinate analysis and permutation multivariate analysis showed that there was a tendency for microbial differentiation between the CON and HG groups (*P* < 0.1), although no significant difference between the HG and HP groups was observed (*P* > 0.05). Compared with the CON diet, the HG diet decreased (*P* < 0.05) the abundance of some probiotic species (e.g., *Sphingomonas* and *Candidatus Arthromitus*) and increased (*P* < 0.05) the abundance of acid-producing microbiota (e.g., *Succiniclasticum*, *Nesterenkonia*, and *Alloprevotella*) in the ileal mucosa. Compared with the HG diet, the HP diet decreased (*P* < 0.05) the abundance of *Alloprevotella* and increased (*P* < 0.05) the abundance of *Mycoplasma* in the ileal mucosa. Furthermore, partial least squares discriminant analysis and orthogonal partial least-squared discriminant analysis indicated that different dietary treatments resulted in different metabolic patterns in the ileal mucosa of the CON, HG, and HP groups. The HG diet altered (VIP > 1 and *P* < 0.05) the metabolic patterns of amino acids, fatty acids, and nucleotides/nucleosides (such as increased amounts of ornithine, tyrosine, *cis*-9-palmitoleic acid, and adenosine) compared with the CON diet. However, 10 differential metabolites (VIP > 1 and *P* < 0.05; including tyrosine, ornithine, and *cis*-9-palmitoleic acid) identified in the HG group exhibited a diametrically opposite trend in the HP group, suggesting that the HP diet could partially eliminate the changes brought upon by the HG diet. Collectively, our findings demonstrate that different diets altered the ileal mucosal microbiota and metabolites and provide new insight into the effects of high-grain diets on the intestinal health of ruminant animals.

## Introduction

Currently, a high-grain (HG) feeding is often used during the breeding of ruminant species as it improves production performance and has economic benefits. A series of studies have revealed fundamental knowledge on the effects of HG diets on the ruminant digestive tract (mainly the rumen and hindgut), including effects on microbial composition and function, metabolism, and epithelial gene expression (Plaizier et al., 2012; Liu et al., 2014; Plaizier et al., 2017; Wang et al., 2021). However, the application of HG diets while ensuring the health of ruminant animals is an important issue that needs to be resolved for the breeding of ruminants.

With the development of lamb breeding programs with increased productivity, feeding of the animals with pelleted concentrates has been promoted and implemented in many modern fattening systems (Islam et al., 2017). Recently, accumulating evidence has indicated that the application of pelleted concentrates is an effective method to avoid selective eating, reduce nutrition wastage during storage and feeding, save labor, and increase rearing profitability (Blanco et al., 2015; Zhong et al., 2018; Li et al., 2021b). Previous studies have demonstrated that pelleted HG diets improve rumen fermentation and amplify the use of simple sugars by rumen microbiota (Trabi et al., 2019), although information regarding the impact of pelleted HG diets on the small intestine is limited.

Intestinal mucosa physically separates the inimical intestinal contents from the host, and also severs as the interface for the crosstalk, such as chemical dialogue through metabolites, between microbiota and their host (Wells et al., 2011; Xie et al., 2013). Previous studies have shown that mucosal host-microbiota metabolic interactions are crucial to intestinal homeostasis and host health (Yuan et al., 2020). For instance, metabolic and microbial changes in the intestinal mucosa of humans with some diseases (such as inflammatory bowel disease) exhibit interdependent and co-directional relationships (McHardy et al., 2013; Ahmed et al., 2016); probiotics such as *bifidobacteria* and *lactobacillus* can inhibit the proliferation of pathogenic bacteria and regulate the intestinal immunity by directly adhering to the intestinal mucosa and their metabolites (He et al., 2001; Hammes and Hertel, 2006). For animals, various factors, especially diets, have important effects on the intestinal microbiota and its metabolites (Wang et al., 2019; Yao et al., 2021). Hence, understanding the different dietary effects on intestinal mucosal microbiota and metabolites would provide favorable information for the subsequent development of dietary probiotics in ruminants. However, limited information is currently available regarding the mucosal microbial and metabolic changes in the small intestine of ruminants under HG or pelleted HG diets.

In the present study, we used the combination of 16S rRNA gene sequencing and liquid chromatography-mass spectrometry (LC/MS) to investigate mucosal microbiota and their metabolic profiles in the ileum of Hu sheep fed low-grain, pelleted or non-pelleted high-grain diets. Our findings contribute to a deeper understanding of the impacts of high-grain diets, and improve our understanding of mucosal host-microbiota metabolic interactions.

## Materials and Methods

### Experimental Design, Animals and Diets

All animal experimentation was conducted in accordance with the Experimental Animal Welfare Ethics Committee of Shenyang Agricultural University. The present study was part of a series of studies designed to evaluate the effects of high-grain diets on the physiological health and microbial community of the gastrointestinal tract, with detailed experimental design, animals and diets described in previous studies (Trabi et al., 2019; Trabi et al., 2020). Briefly, a total of 15 male Hu-breed lambs, initial body weight (26.80 ± 0.32 kg), were randomly assigned to a control diet (non-pelleted low-grain diet; CON; *n* = 5), a non-pelleted high-grain diet (HG; *n* = 5), or a pelleted high-grain diet (HP; *n* = 5). The CON group were fed a control diet containing 30% mashed concentrate and 70% forage, while the HG group were fed a high-grain diet containing 70% mashed concentrate and 30% forage. The ingredients of the HP diet were same as the HG diet, but in pelleted form. The pelleting process was conducted using a pelleting machine (Jiangsu Muyang Group Co., Ltd., China) with a diameter of 3.2 mm. All sheep were placed in individual pens (1.2 m × 1.2 m), and experimental diets and water were supplied *ad libitum* throughout the 60-day experimental period. Ingredients and nutritional compositions of the experimental diets were presented in Supplementary Table 1.

### Sample Collection

On day 60 of treatment, animals from each treatment group were euthanized by captive bolt stunning at 4–5 h after the morning feeding. Immediately after slaughter and dissection, ileum tissue was washed with ice cold sterile phosphate-buffered saline until no digesta was visible, then mucosal samples were scraped using sterile slides. Ileal mucosal samples were homogenized and mixed thoroughly, with a representative sample collected into sterile tubes, flash frozen in liquid nitrogen, and stored at −80°C until DNA extraction and metabolome measurement.

### DNA Extraction, Illumina Sequencing, and Bioinformatics Analysis

Extraction of total genomic DNA from the ileal mucosa was performed following the standard protocol of the QIAamp DNA stool Mini Kit (QIAGEN, Hilden, Germany). The integrity and concentration of extracted DNA was measured using 1% agarose gel electrophoresis and a NanoDrop spectrophotometer (Nyxor Biotech, Paris, France). The hypervariable V3-V4 region in the bacterial 16S rRNA of all samples were targeted for PCR amplification using the bacterial universal primers 338F (5′-ACTCCTRCGGGAGGCAGCAG-3′) and 806R (5′-GGACTACCVGGGTATCTAAT-3′) (Mao et al., 2015). After amplification, PCR products from 3 replicates for each sample were mixed and then purified using the AxyPrep DNA Gel Extraction Kit (Axygen Biosciences, Union City, CA, United States). 16S rRNA sequencing was performed on an Illumina Miseq platform using standard protocols.

Raw sequence reads were demultiplexed and quality-filtered using Quantitative Insights into Microbial Ecology (QIIME) version 1.8.0-dev (Caporaso et al., 2010). Paired-end reads were merged using FLASH (version 1.2.7) (Magoc and Salzberg, 2011), with a minimum overlap of 10 bp and a maximal expected error of 0.2. Harvested sequences were clustered into OTUs at a similarity level of 97% using UPARSE (version 7.1^1^) (Edgar, 2013) and chimeras were removed using UCHIME (Edgar et al., 2011). The taxonomic analysis on the OTU representative sequences was performed using the Ribosomal Database Project (RDP) classifier (Wang et al., 2007), and the SILVA database (version 119) was used for comparisons of the 16S bacteria (DeSantis et al., 2006). Richness and diversity of the microbial communities were reflected by indices such as Chao 1, Shannon index, and Simpson. Sequence data generated in the current research had been deposited into the Sequence Read Archive (SRA) database under accession no. PRJNA734004.

### Liquid Chromatography-Mass Spectrometry (LC/MS) Analysis

Prior to LC-MS analysis, 50 mg of mucosa sample was transferred into a 1.5-mL Eppendorf tube, mixed with 800 μL methanol by ultrasonication for 90 s at 65 Hz at 4°C for 10 min, and then centrifuged at 12000 rpm at 4°C for 15 min. Finally, after a series of pretreatments, 200 μL of the supernatant and 5 μL DL-o-Chlorophenylalanine (0.14 mg/ml, as interior label) were transferred into the injection vial for the LC/MS analysis. Metabolomic analysis was conducted using a LC-MS system (Waters, UPLC; Thermo, Q Exactive) with a Waters Acquity UPLC HSS T3 column (100 mm × 2.1 mm, 1.8 μm). The column temperature was maintained at 40°C, and the flow rate was set to 0.3 mL/min. The mobile phase A was water with 0.05% (v/v) formic acid, and the mobile phase B was acetonitrile. During measurement, the injection volume of each sample was 6 μL, and the automatic injector temperature was set as 4°C.

The LC-MS system was equipped with both positive electrospray ionization (ESI+) and negative electrospray ionization (ESI-) modes. The measurement parameters for ESI+ mode were: Heater temperature 300°C; Sheath gas flow rate 45 arb, aux gas flow rate 15 arb, sweep gas flow rate 1 arb, spray voltage 3.0 KV, capillary temperature 350°C and S-Lens RF level 30%. Spray voltage and S-Lens RF level for the ESI- mode were set to 3.2 KV and 60%, with the other parameters the same as used for the ESI+ mode. Raw data was extracted and preprocessed using Compound Discoverer 2.0 software (Thermo), and normalized according to the interior label using Excel 2010 software. Harvested data included sample names, peak intensity, retention time (RT) and compound molecular weight, and metabolite identification was performed using the Kyoto Encyclopedia of Genes and Genomes Database^2^ and the online Human Metabolome Database^3^.

### Statistical Analysis

All statistical analysis of the experimental data was performed using SPSS software (SPSS v. 20.0, SPSS Inc., Chicago, IL, United States). Differences among the microbial communities from the different experimental groups were detected by a principal coordinate analysis (PCoA) and the permutation multivariate analysis (PERMANOVA). Significance of the microbial differences at the phylum and genus level were identified using a Wilcoxon-Mann-Whitney *U* test. Featured OTUs in each experimental group were identified using a linear discriminant analysis effect size (LEfSe) analysis.

Normalized peak intensities of the metabolites were imported into SIMCA-P + 14.1 software (Umetrics, Umea, Sweden) for partial least squares discriminant analysis (PLS-DA) and orthogonal partial least-squared discriminant analysis (OPLS-DA). Metabolites at different abundances between groups were validated using the Variable importance in Projection (VIP) generated from PLS-DA and a Wilcoxon-Mann-Whitney *U* test (VIP > 1 and *P* < 0.05). Spearman rank correlation coefficients (r) between the differential metabolites and OTUs were calculated using a R program with a significance threshold of | *r*| > 0.6 and *P* < 0.05 adopted, and visualization of the correlation network was performed using Gephi 0.8.2 software^4^.

## Results

### General Sequencing Observations

**FIGURE 1:**
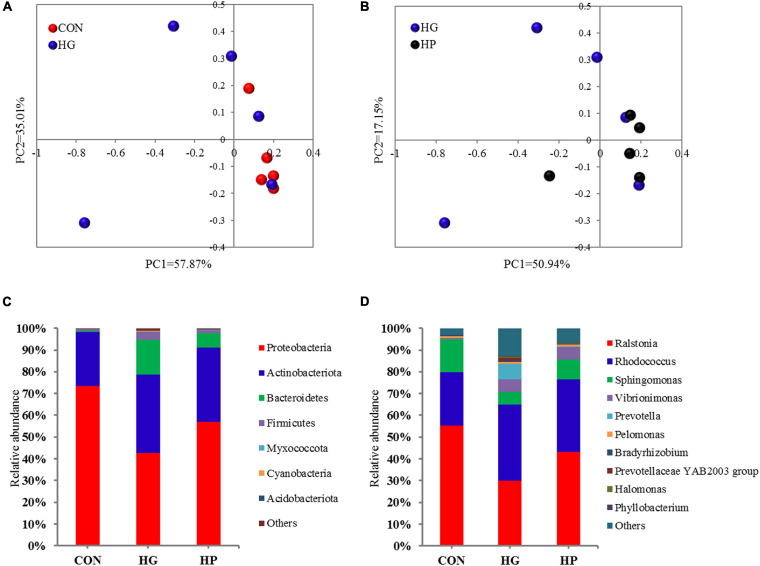
Taxonomic analysis of the microbial communities in the ileal mucosa of Hu sheep fed CON, HG, and HP diets. Principal coordinate analysis (PCoA) score plots of the samples from the CON and HG group **(A)** and the HG and HP group **(B)** based on the Bray-Curtis similarity metric. The composition of the ileal mucosal microbiota at the phylum **(C)** and genus **(D)** levels.

In the present study, a total of 696,525 valid V3-V4 16S rRNA reads were obtained for further analysis after sequencing, with an average of 46,435 sequences per sample. Rarefaction curves of the ileal mucosal microbiota were conducted, and the results showed that the samples provided sufficient sequences to detect most of the microbial species (Supplementary Figure 1). Estimates of microbial richness and diversity showed that no significant differences were observed among the different dietary treatments (Supplementary Table 2). Results of the PCoA (Figures 1A,B) combined with PERMANOVA illustrated that there were certain microbial differences between the CON and HG groups (*P* = 0.082), with no significant differences between the HG and HP groups (*P* = 0.662).

### Alterations in Microbial Composition of the Ileal Mucosa After Different Dietary Treatments

To further evaluate the effects of different diets on ileal mucosal microbiota, we conducted a statistical analysis at different microbial taxonomic levels. At the phylum level (Figure 1C), the phyla Proteobacteria (average 57.80%), Actinobacteriota (average 31.46%), Bacteroidetes (average 7.74%), and Firmicutes (average 1.96%) occupied dominant positions for all three dietary groups. The relative abundance of Bacteroidetes tended to increase in the HG group compared to the CON group (*P* = 0.056), while the other phyla had no significant differences among the CON, HG, and HP groups (*P* > 0.05). At the genus level (Figure 1D), regardless of dietary treatment, *Ralstonia* (average 42.82%), *Rhodococcus* (average 30.87%), *Sphingomonas* (average 10.06), *Vibrionimonas* (average 3.97), and *Prevotella* (average 2.51%) were the most dominated genera in the ileal mucosal microbiota. As for genus-specific differences (Table 1), a significant decrease (*P* < 0.05) in the relative abundance of *Sphingomonas*, *Candidatus Arthromitus*, and [Eubacterium] coprostanoligenes group_norank, and a significant increase (*P* < 0.05) in the relative abundance of *Succiniclasticum*, *Nesterenkonia*, *Alloprevotella*, *Labrys*, and *Paracoccus*, were observed in the HG group compared with the CON group. In addition, a lower relative abundance of *Alloprevotella* (*P* < 0.05) and a higher relative abundance of *Mycoplasma* (*P* < 0.05) were observed in the HP group compared with the HG group.

**TABLE 1:** Relative abundance of major genera (>0.01% in at least one group) in the ileal mucosal microbiota of Hu sheep fed CON, HG, and HP diets.

**Items**	**Treatments**			**SEM**	***P* value**	
	**CON**	**HG**	**HP**		**CON vs. HG**	**HG vs. HP**
*Sphingomonas*	15.382	5.798	8.989	1.435	0.032	0.222
*Candidatus Arthromitus*	18.255	0.950	5.302	0.101	0.008	1.000
[Eubacterium] coprostanoligenes group_norank	0.003	0.116	0.057	0.027	0.016	0.421
*Succiniclasticum*	0.001	0.099	0.025	0.019	0.008	0.056
*Nesterenkonia*	0.004	0.073	0.022	0.014	0.016	0.310
*Alloprevotella*	<0.001	0.069	<0.001	0.019	0.032	0.032
*Mycoplasma*	0.023	0.001	0.028	0.006	0.222	0.008
*Labrys*	0.001	0.024	0.014	0.006	0.008	0.095
*Paracoccus*	<0.001	0.012	0.003	0.002	0.032	0.151

**FIGURE 2:**
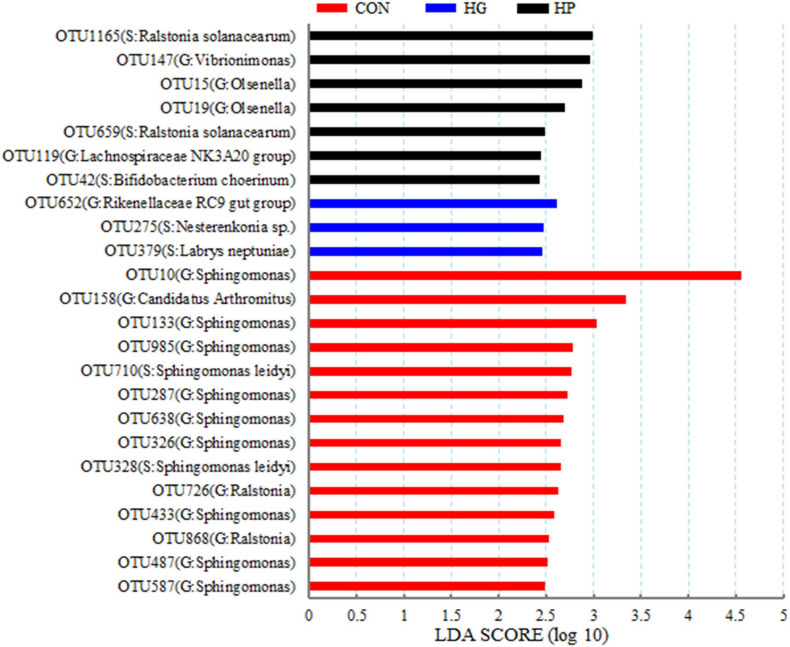
Linear discriminant analysis (LDA) scores of the microbial OTUs in the ileal mucosa of Hu sheep fed CON, HG, and HP diets.

At the OTU level, a LEfSe analysis was performed to identify the featured microbial species in the different dietary treatments (Figure 2). Our results showed that 24 OTUs were significantly affected by dietary treatment (*P* < 0.05), and these OTUs accounted for 11.63, 0.09, and 0.50% of the total sequences in the CON, HG, and HP groups, respectively. Briefly, 14 OTUs (11 OTUs belonging to the genus *Sphingomonas*, 2 OTUs belonging to the genus *Ralstonia*, and 1 OTU belonging to the genus *C. Arthromitus*) were featured in the CON group (*P* < 0.05). For the HG group, OTU379 (S: *Labrys neptuniae*), OTU275 (S: *Nesterenkonia* sp.), and OTU652 (G: *Rikenellaceae RC9 gut group*) were the featured ileal mucosal microbiota (*P* < 0.05). Moreover, OTU42 (S: *Bifidobacterium choerinum*), OTU119 (G: *Lachnospiraceae NK3A20 group*), OTU659 and OTU1165 (S: *Ralstonia solanacearum*), OTU19 and OTU15 (G: *Olsenella*), and OTU147 (G: *Vibrionimonas*) were featured in the HP group (*P* < 0.05).

### Changes in the Metabolic Profiles of Ileal Mucosa After Different Dietary Treatments

**FIGURE 3:**
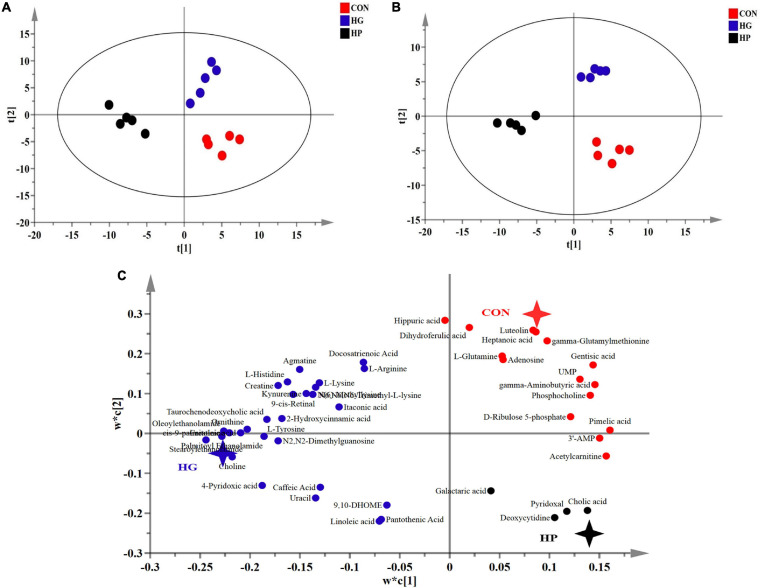
Partial least squares discriminant (PLS-DA) (**A**, score plots; **C**, loading plots) and orthogonal partial least squares discriminant (OPLS-DA; **B**) analysis of the ileal mucosal metabolites from Hu sheep fed CON, HG, and HP diets.

**FIGURE 4:**
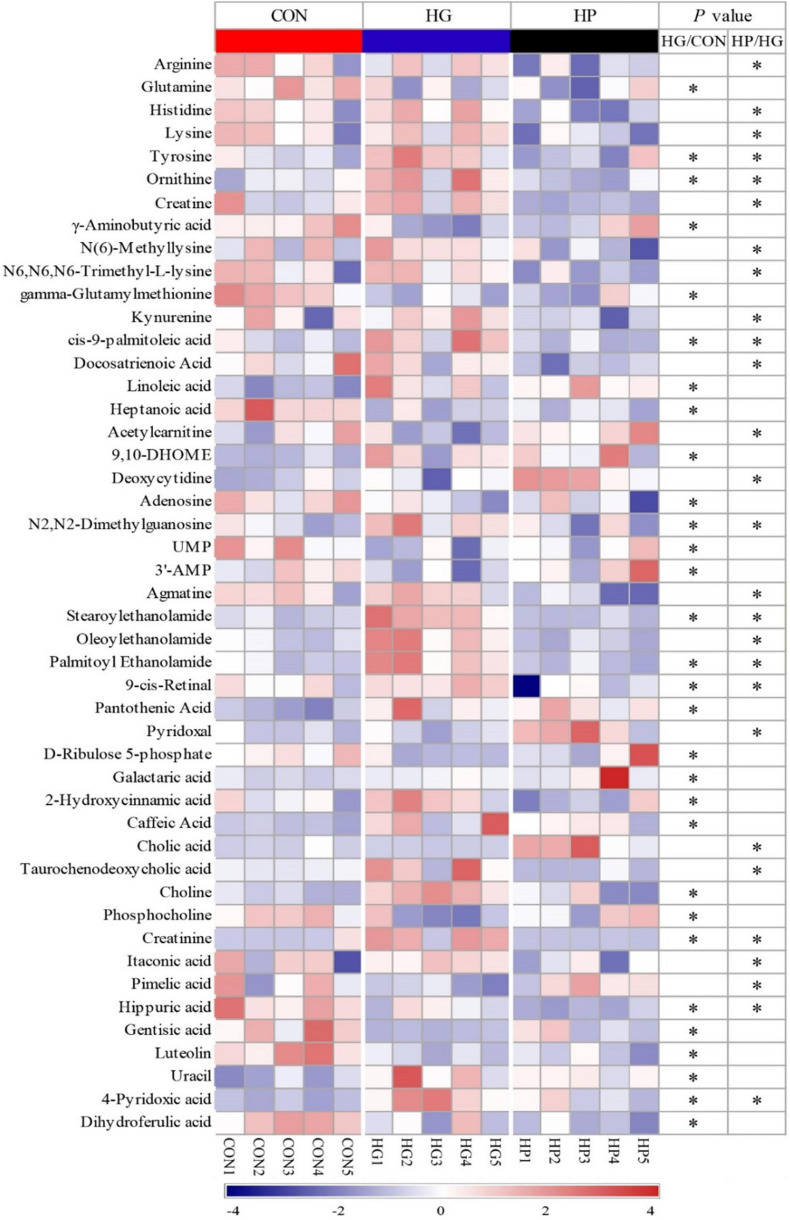
Heat-map visualizing of the differential metabolites identified in the ileal mucosal samples of sheep fed CON, HG, and HP diets.

To determine whether changes in microbial composition resulted in metabolic alterations, a LC-MS based metabolome analysis was used to reveal the metabolic profiles of the ileal mucosa with the different diets. After quality control and identification, a total of 238 reliable metabolites were found in the mucosal samples, which included organic acids, amino acids and their derivatives, fatty acids, sugars and their derivatives, amines, nucleoside, and others. PLS-DA and OPLS-DA score plots (Figure 3) of the identified metabolites in the mucosal samples revealed a clear separation between the CON and HG groups, as well as between the HG and HP groups, indicating that the different dietary treatments had a great impact on the mucosal metabolites. Moreover, the PLS-DA loading plots showed that the distribution of the important mucosal metabolites (VIP > 1.0). To assess the specific effects of the dietary treatments on mucosal metabolites, VIP values obtained from the PLS-DA analysis, combined with the statistical analysis, were used to screen for differential metabolites. In total, 47 metabolites (VIP > 1.0 and *P* < 0.05) were significantly affected by dietary treatments (Figure 4). In general, these results revealed that the major differential metabolites among the different dietary treatments were amino acids and their derivatives, fatty acids, and nucleosides/nucleotides, followed by amines, sugars and their derivatives, and others.

For amino acids and their derivatives (Figure 4), the HG diet significantly increased (*P* < 0.05) the content of ornithine and tyrosine, while significantly decreasing (*P* < 0.05) the content of glutamine and γ-aminobutyric acid compared with the CON diet. HP diet significantly decreased (*P* < 0.05) the content of arginine, histidine, lysine, tyrosine, ornithine, creatine, N(6)-Methyllysine, and N6, N6, N6-Trimethyl-L-lysine compared with the HG diet. Additionally, HG diet significantly increased (*P* < 0.05) the content of stearoylethanolamide, and palmitoyl ethanolamide compared with the CON diet, while HP diet significantly decreased (*P* < 0.05) the content of agmatine, stearoylethanolamide, oleoylethanolamide, and palmitoyl ethanolamide compared with the HG diet. Moreover, a significant increase in the content of choline and a significant decrease in the content of phosphocholine were observed in the HG group compared with the CON diet.

For fatty acids, a significant increase (*P* < 0.05) in the content of *cis*-9-palmitoleic acid, linoleic acid, and 9,10-DHOME, and a significant decrease (*P* < 0.05) in the content of heptanoic acid, were observed in the HG group compared with the CON group. A significant decrease (*P* < 0.05) in the content of *cis*-9-palmitoleic acid and docosatrienoic acid were observed in the HP group compared with HG group. Meanwhile, among metabolites related to lipid digestion, HP diet substantially increased the content of cholic acid (*FC* = 30.28, *P* < 0.05) and decreased the content of taurochenodeoxycholic acid (*FC* = 0.13, *P* < 0.05) in the ileal mucosa compared with HG group. For nucleosides/nucleotides, HG diet significantly increased the content of N2,N2-dimethylguanosine, while decreasing (*P* < 0.05) the content of adenosine, UMP, and 3′-AMP compared with the CON diet. HP diet significantly increased (*P* < 0.05) the content of deoxycytidine, and significantly decreased (*P* < 0.05) the content of N2,N2-dimethylguanosine compared with the HG diet.

**FIGURE 5:**
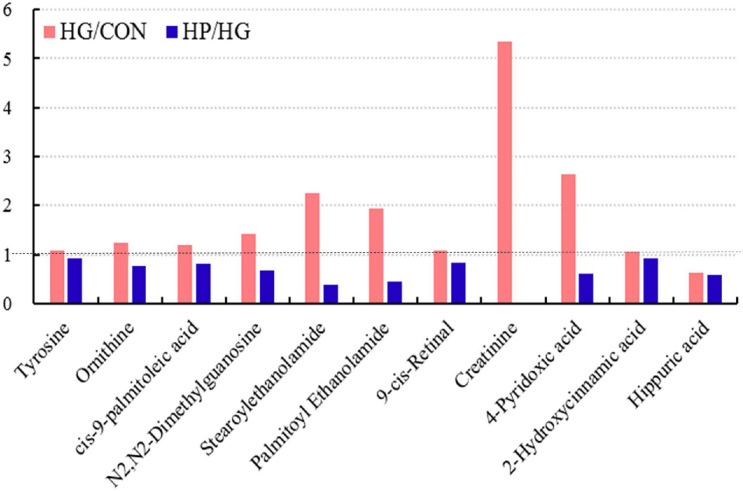
Common differential metabolites in the ileal mucosa of Hu sheep fed CON, HG, and HP diets.

Interestingly, our results also found that a total of 11 differential metabolites were simultaneously identified in the HG group (compared with the CON group) and the HP group (compared with the HG group), with the fold change of these common differential metabolites presented in Figure 5. These results showed that, except for hippuric acid, the fold change of these 10 metabolites (tyrosine, ornithine, *cis*-9-palmitoleic acid, N2,N2-Dimethylguanosine, stearoylethanolamide, palmitoyl ethanolamide, 9-*cis*-Retinal, creatinine, 4-pyridoxic acid, and 2-hydroxycinnamic acid) in the HG and HP groups were exhibited a diametrically opposite directions. It seems that the HP diet partially eliminates the changes brought on by the HG diet.

### Mucosal Host-Microbiota Metabolic Interactions in the Ileum

**FIGURE 6:**
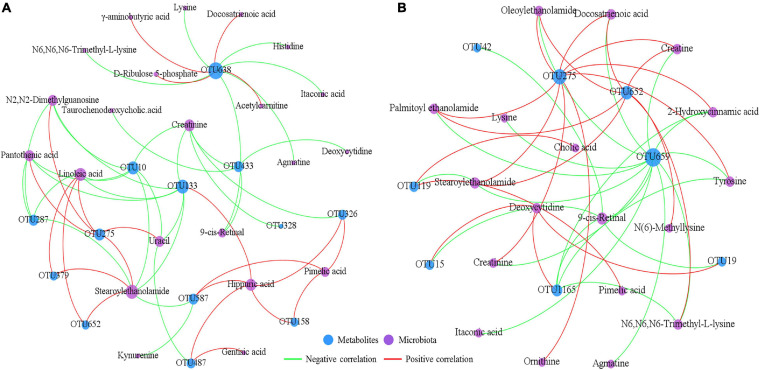
Correlation networks between the affected microbiota and metabolites in the ileal mucosa of Hu sheep between the CON and HG groups **(A)** and the HG and HP groups **(B)**. Only correlation coefficients with *P* < 0.05 and the |*r*| > 0.6 were adopted to construct the correlation networks.

To explore the potential mucosal host-microbiota metabolic interactions in the ileum, a correlation network analysis was conducted using the disrupted microbial OTUs and the differential metabolites identified in the different dietary treatments (Figure 6). The results of this analysis demonstrated that there were complex connections between the intestinal mucosal microbiota and the metabolites. For example, the correlation network constructed using data from the CON and HG groups showed that OTU638 (G: *Sphingomonas*) was positively correlated with γ-aminobutyric acid, D-ribulose 5-phosphate, acetylcarnitine and docosatrienoic acid, and negatively correlated with N6,N6,N6-trimethyl-L-lysine, itaconic acid, lysine, histidine, agmatine, and 9-*cis*-retinal. The correlation network constructed using data from the HG and HP groups showed that OTU659 (S: *R. solanacearum*) was negatively correlated with 13 metabolites (such as oleoylethanolamide, tyrosine, creatine, lysine, and agmatine), while OTU275 (S: *Nesterenkonia* sp.) was positively correlated with eight metabolites (such as creatinine, 2-hydroxycinnamic acid, ornithine, and tyrosine).

## Discussion

Acting as the biological barrier for the intestine, mucosal microbiota play an important role in resisting invasion by pathogenic microbiota and by regulating host immunity (Ashida et al., 2012). Extensive studies have recently been conducted on mucosal microbiota and the remodeling effect of HG diet in the rumen and hindgut of ruminant animals (Chen et al., 2011; Ye et al., 2016; Wang et al., 2017; Zhang et al., 2017). However, studies on the mucosal microbiota of the ileum and the effects of HG or HP diets on them are limited. At present, 16S rRNA sequencing with an Illumina MiSeq Platform provides an efficient mean to study the intestinal microbiota (Rapin et al., 2017), and also has been widely used in ruminant researches (Li et al., 2018; Lin et al., 2020). Through sequencing, our results provide an overview of the microbial composition of the ileal mucosa of the Hu sheep. Consistent with other studies using 16S rRNA sequencing, our results found that the dominant bacterial phyla in ileal mucosal microbiota were Proteobacteria, Actinobacteriota, Bacteroidetes, and Firmicutes (Mao et al., 2015; Liu et al., 2019). As for genus level, our results indicated that *Ralstonia*, *Rhodococcus*, *Sphingomonas*, and *Vibrionimonas* dominated in ileal mucosal microbiota, while this result showed great differences from other studies (Mao et al., 2015; Liu et al., 2019). The reason for this discrepancy may be related to different dietary composition, animal age, and host species.

Microbial disturbance caused by the HG diets in the rumen has been confirmed by numerous studies (Zhang et al., 2017; Seddik et al., 2019), but the results of the studies on the effect of HG diet on the ileal microbiota in ruminants is inconsistent. Liu et al. (2019) reported that HG diets resulted no significant between-group differences in the composition of ileal mucosal microbiota in goats, while Jiao et al. (2018) revealed dissimilarities in the ileal mucosal microbiota between their HG and CON groups. In our present study, the results of PCoA and PERMANOVA analysis showed that there was a tendency for a microbial difference between the CON and HG groups, while no significant difference between the HG and HP groups was observed. Hence, the above results indicate that the impact of HG diet on the rumen is greater than that on the ileum, and more studies are needed to reveal the exact reason for the discrepancy.

To further evaluate the specific differences in the microbial communities between the different dietary treatments, we assessed microbial differences at the genus and OTU levels. Our results revealed that the relative abundance of the genus *Sphingomonas*, and several species (e.g., OTU587, OTU487, and OTU433) significantly declined in the ileal mucosal microbiota of the HG group compared with the CON group. *Sphingomonas* is a strictly aerobic commensal bacteria of the human and animal intestines, and existing evidence from studies suggests that *Sphingomonas* contributes to the degradation of several types of polysaccharides, such as cellulose (Birkholz and Kronenberg, 2015; Cui et al., 2020). Moreover, *Sphingomonas* spp. is used as a probiotic in intensive aquaculture, and is reported to have antagonistic effects on fish pathogens (Chaudhary and Qazi, 2014). Hence, the higher abundance of *Sphingomonas* in the CON group may be linked with its cellulose degradation activity and exerts a probiotic effects in the small intestine of the Hu sheep. Our results also found that the relative abundance of the genus *C. Arthromitus* and its species—OTU158 were significantly decreased in the ileal mucosal microbiota of the HG group compared with the CON group. *C. Arthromitus* is a Gram positive spore-forming segmented filamentous bacteria that colonizes the ileal mucosa of mammals (Caselli et al., 2010; Thompson et al., 2013). Previous studies showed that *C. Arthromitus* plays vital roles in the immune system maturation, defense against pathogenic infection, and maintenance of immune balance of the intestinal mucosa (Caselli et al., 2010; Schnupf et al., 2013). Hence, reduction of *C. Arthromitus* caused by the HG diets in the present study may be detrimental to intestinal mucosal health and leave the hosts more susceptible to infectious diseases.

In addition, results of the present study revealed that HG diets increased the relative abundance of some acid-producing microbiota, such as *Succiniclasticum*, *Nesterenkonia*, and *Alloprevotella*. Of these genera, *Succiniclasticum* spp. can convert succinate, produced by other microorganisms fermenting carbohydrates, to propionate (Van Gylswyk, 1995). *Nesterenkonia* strains have the ability to secrete amylase, and hence produce alcohols such as acetone, butanol, and ethanol, as well as acetate and butyrate under aerobic conditions (Ebrahimi et al., 2020). Members of *Alloprevotella* possess saccharolytic activity, and produce moderate amounts of acetate and major amounts of succinate as end products of fermentation (Downes et al., 2013). Thus, similar to the rumen, changes in theses acid-producing microbiota in the HG group is likely related to the increased levels of starch in their diet, and the altered microbial community may result an acidic microenvironment in the ileal mucosa. Interestingly, in contrast to the reduced abundance of *Alloprevotella* in the HG group, the HP group significantly decreased the relative abundance of this genus in the present study. As for other acid-producing microbiota, OTU42, belonging to *B. choerinum*, was found to be significantly enriched in the HP group, but not in the CON or HG groups. *Bifidobacterium* sp., an anaerobic probiotic species that colonizes animal intestines, plays an important role in inhibiting the proliferation of pathogenic bacteria and maintaining intestinal immune health (Wang et al., 2020). Members of *Bifidobacterium* can utilize a diverse range of dietary carbohydrates that evade rumen degradation and their presence in high numbers often has beneficial effects on the health of the intestine and the host (Pokusaeva et al., 2011; Bunesova et al., 2014).

To further understand the effects of HG and HP diets on the ileal mucosa, we conducted a LC-MS based metabolome analysis. Interestingly, contrary to the minor fluctuations of mucosal microbiota observed above, dietary treatments had large effects on mucosal metabolites. Our results showed that samples from the CON, HG, and HP groups were clearly separated on the PLS-DA and OPLS-DA scatter plots, indicating that the different dietary treatments resulted in different metabolic patterns in the ileal mucosa. For specifically affected metabolites, the HG diets increased the content of ornithine and tyrosine, and decreased the content of glutamine and γ-aminobutyric acid compared with the CON diet. The increased amino acids should be available to the nutrition of the host, but also increase potential risks for the intestines. In ruminants, ornithine and tyrosine are precursors of putrescine and tyramine, with these corresponding biogenic amines generated by decarboxylation of amino acids through microbial activity (Saleem et al., 2012). The higher content of ornithine and tyrosine may result in more accumulation of biogenic amines as is observed in the rumen (Xue et al., 2018), which might increase intestinal pathological processes such as cell apoptosis and inflammation (Linares et al., 2016; Zhang et al., 2017). However, due to the limitations of LC-MS technology, putrescine and tyramine were not detected in the present study, but other amines, including stearoylethanolamide palmitoyl ethanolamide, were observed to have a significant increase in the HG group. Simultaneously, contrary to the alterations in the HG group, the HP group significantly decreased the content of several amino acids (such as arginine, histidine, and ornithine) and amines (such as agmatine, stearoylethanolamide, and oleoylethanolamide) compared with the HG group. In addition, glutamine serves as important energy source for the intestinal mucosa, mediates multiple protective influences on intestinal homeostasis, such as promoting cell proliferation and differentiation, maintaining intestinal mucosal integrity, and protecting the physical barrier of the intestine (Rao and Samak, 2012). Moreover, as a nutritional regulatory factor, γ-aminobutyric acid can be converted into glutamine, and then exert its beneficial effects (Chen et al., 2015). Therefore, the reduced content of glutamine and γ-aminobutyric acid in HG groups may imply that the ileum is more fragile after feeding with the HG diet and can easily acquire damage to integrity and barrier function of the ileum. This speculation is supported by the study of Liu et al. (2019), who reported that HG diets caused downregulated mRNA expression of several tight junction genes (e.g., claudin-4, occludin, and ZO-1) in the ileal mucosa of the goat (Liu et al., 2019).

Besides amino acids and amines, results of our study on nucleotides/nucleosides also provided evidence that the HG diets were not conducive to intestinal health. In our results we found three nucleotides/nucleosides, adenosine, UMP, and ′-AMP, which had significantly decreased amounts in the ileal mucosa of the HG group compared with the CON group. Nucleotides and nucleosides have been reported to have multiple biological effects on intestinal health, such as enhancing cell proliferation and differentiation, improving intestinal morphology, and repairing tissue damage (Sato et al., 1999; Vieites et al., 2008; Rodríguez-Serrano et al., 2010). In addition, luteolin, a natural antioxidant, was observed to be significantly decreased in the HG group in our study. As a member of the flavonoid family, luteolin widely exists in many plants and has various beneficial anti-inflammatory and anti-oxidative functions (Li et al., 2021a). Previous studies have suggested that HG diets increase the potential risk of intestinal inflammation and damage to the mucosal barrier (Jiao et al., 2018; Liu et al., 2019), thus, decreased nucleotide/nucleoside and luteolin content might imply that HG diets weakened the ability of self-renewal, inhibiting inflammation, and self-repair.

Bile acids, synthesized in hepatocytes via oxidation of cholesterol and re-absorbed in the ileum after being modified by the intestinal microbiota, are critically important for lipid metabolism, regulating intestinal microbial balance, and promoting immune homeostasis (Jia et al., 2018). Interestingly, significant differences in the two primary bile acids (cholic acid and taurochenodeoxycholic acid) were observed in ileal mucosa between the HG and HP groups in the present study. A previous study demonstrated that HG diets altered the bacterial communities in the jejunum and significantly decreased the content of cholic acid in bile from cattle (Liu et al., 2020). Another study revealed that high rumen-degradable starch diets significantly increased the hepatic concentration of taurochenodeoxycholic acid in the liver compared with the control group (Shen et al., 2020). In the present study, the same trend (increased taurochenodeoxycholic acid and decreased cholic acid) was found in the ileal mucosa of the HG group, but it did not reach a statistically significant level. However, the opposite trend was observed in the ileal mucosa of the HP group. It is worth noting that, *Bifidobacterium* spp. has the ability to secrete bile salt hydrolases to deconjugate the amide bond between bile acids with glycine or taurine (Urdaneta and Casadesús, 2017), hence, the increased relative abundance of *Bifidobacterium* spp. is a reasonable explanation for the decreased levels of taurochenodeoxycholic acid in the HP group in the present study. Corresponding to the changes in the bile acids in the HP group, a significant decrease in two fatty acids (*cis*-9-palmitoleic acid and docosatrienoic acid) and a significant increase in acetylcarnitine were observed in the HP group in the present study. Acetylcarnitine serves as effective carriers to transport long-chain fatty acids into mitochondria for β-oxidation to meet the energy requirements of cells (Li et al., 2019). Therefore, decreased content of fatty acids in the HP group may be related to the effects of bile acids and acetylcarnitine in promoting lipid metabolism. The precise mechanisms for bile acids and lipid metabolism after feeding with HG and HP diets need additional systematic study.

## Conclusion

In summary, the present study integrated microbiome and metabolome analyses to demonstrate that HG diets cause substrate-dependent alterations in the mucosal microbiota and metabolites. Ileal mucosal microbiota, such as the probiotics (*Sphingomonas* and *C. Arthromitus*) decreased and the acid-producing microbiota (*Succiniclasticum*, *Nesterenkonia*, and *Alloprevotella*) increased, and further alterations in the metabolism of amino acids, fatty acids and nucleotides/nucleosides were observed in the ileal mucosa of Hu sheep fed a HG diet. This mucosal host-microbiota metabolic interaction causes the ileal mucosa of the HG group to be more likely exposed to an unfriendly environment with increased risk of potential inflammation. However, HP diet seemed to partially reverse these changes caused by the HG diet. Our results help to better understand the effects of high-grain diets on health of ruminant species and their application in animal production.

## Data Availability Statement

The datasets presented in this study can be found in online repositories. The names of the repository/repositories and accession number(s) can be found in the article/Supplementary Material.

## Ethics Statement

The animal study was reviewed and approved by the Experimental Animal Welfare Ethics Committee of Shenyang Agricultural University.

## Author Contributions

SZ, SM, and RZ designed the study. LL, FX, HM, and ZZ conducted the experiment and collected the samples. HM and ZZ conducted laboratory analyses. RZ analyzed the data and wrote the manuscript. ZW, SM, DI, and SZ revised the manuscript. All authors contributed to the article and approved the submitted version.

## Conflict of Interest

The authors declare that the research was conducted in the absence of any commercial or financial relationships that could be construed as a potential conflict of interest.

## Publisher’s Note

All claims expressed in this article are solely those of the authors and do not necessarily represent those of their affiliated organizations, or those of the publisher, the editors and the reviewers. Any product that may be evaluated in this article, or claim that may be made by its manufacturer, is not guaranteed or endorsed by the publisher.

## Footnotes

1
http://drive5.com/uparse/

2
http://www.genome.jp/kegg

3
http://www.hmdb.ca

4
https://gephi.org/
