# Supplementary material for: Mucosal Microbiota and Metabolome in the Ileum of Hu Sheep Offered a Low-Grain, Pelleted or Non-pelleted High-Grain Diet
Source: Front Microbiol. 2021 Aug 26;12:718884. doi: 10.3389/fmicb.2021.718884 (PMC8427290; doi:10.3389/fmicb.2021.718884)

Table S1 Ingredients, proximate analysis, and nutrients intake of low-grain diet (CON), mashed (HG) and pelleted (HP) high-grain diets.

| Item | CON | HG | HP |
| --- | --- | --- | --- |
| **Ingredients,(g/kg)DM** |  |  |  |
| Oat straw | 520 | 230 | 230 |
| Alfalfa hay | 180 | 70.0 | 70.0 |
| Corn | 192 | 406 | 406 |
| Wheat bran | - | 156 | 156 |
| Soybean meal | 67.0 | 90.0 | 90.0 |
| Stone powder | 5.00 | 17.5 | 17.5 |
| Calcium hydrogen phosphate | 9.00 | 3.50 | 3.50 |
| Zeolite powder | 10.0 | 10.0 | 10.0 |
| Salt | 7.00 | 7.00 | 7.00 |
| Trace mineral salt and vitamins ^a^ | 10.0 | 10.0 | 10.0 |
| **Nutrient composition** |  |  |  |
| Metabolic energy (MJ/kg) ^b^ | 8.92 | 9.89 | 9.89 |
| Crude protein (g/kg） | 139 | 138 | 138 |
| Neutral detergent fibre (g/kg) | 406 | 267 | 265 |
| Acid detergent fibre (g/kg) | 260 | 147 | 146 |
| Indigestible neutral detergent fibre (g/kg) | 182 | 123 | 123 |
| Indigestible acid detergent fibre (g/kg) | 114 | 77.6 | 75.6 |
| Nitrogen fractions associated to the fibre (g/kg) | 52.0 | 44.0 | 42.9 |
| Ether extract (g/kg) | 36.0 | 34.6 | 33.4 |
| Ash (g/kg) | 81.8 | 64.5 | 65.2 |
| **Nutrients intake** |  |  |  |
| Metabolic energy (MJ/animal/day) | 133 | 152 | 139 |
| Crude protein (g/animal/day) | 207 | 213 | 194 |
| Neutral detergent fibre (g/animal/day) | 607 | 413 | 373 |
| Acid detergent fibre (g/animal/day) | 390 | 227 | 205 |
| Ether extract (g/animal/day) | 53.5 | 53.1 | 46.6 |
| Ash (g/animal/day) | 122 | 98.9 | 90.9 |

^a^Trace mineral salt and vitamins were purchased from Continental Grain Crop. (Nanjing, China) and contained (per kg of premix) 22,000 IU vitamin A, 5000 IU vitamin E, 100 mg Fe, and 20 mg Cu.

^b^Calculated value.

Table S2 Summary overview of the estimated diversity and richness indexes of the ileal mucosal microbiota of Hu sheep fed CON, HG, and HP diets.

| Parameters | Treatments | | | SEM | *P* value | |
| --- | --- | --- | --- | --- | --- | --- |
|  | CON | HG | HP |  | CON vs. HG | HG vs. HP |
| Coverage, % | 99.79 | 99.68 | 99.7 | 0.03 | 0.548 | 1 |
| Chao 1 | 469 | 592.8 | 571 | 57 | 0.29 | 0.31 |
| Shannon | 2.12 | 2.58 | 2.28 | 0.15 | 0.151 | 0.548 |
| Simpson | 0.25 | 0.22 | 0.24 | 0.02 | 1 | 0.762 |

Figure S1 Flattened rarefaction curves of the ileal mucosal microbiota of Hu sheep fed CON, HG, and HP diets.


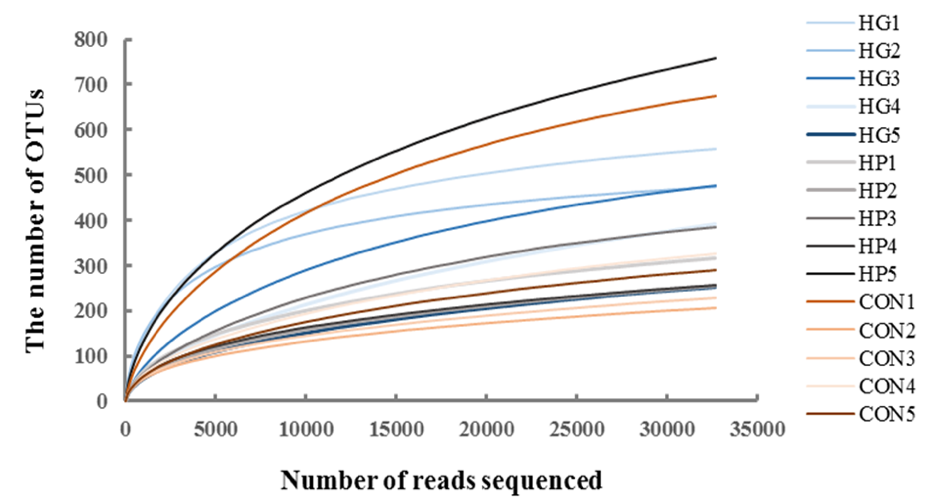

Supplement: Supplementary file 1 [file Table_1.DOCX]
